# Farming legumes in the pre-pottery Neolithic: New discoveries from the site of Ahihud (Israel)

**DOI:** 10.1371/journal.pone.0177859

**Published:** 2017-05-24

**Authors:** Valentina Caracuta, Jacob Vardi, Ytzhak Paz, Elisabetta Boaretto

**Affiliations:** 1 Max Planck-Weizmann Center for Integrative Archaeology and Anthropology, Rehovot, Israel; 2 D-REAMS Radiocarbon Laboratory, Rehovot, Israel; 3 Israel Antiquity Authority, Jerusalem, Israel; New York State Museum, UNITED STATES

## Abstract

New discoveries of legumes in the lower Galilee at the prehistoric site of Ahihud in Israel shed light on early farming systems in the southern Levant. Radiocarbon dating of twelve legumes from pits and floors indicate that the farming of legumes was practiced in southern Levant as early as 10.240–10.200 (1σ) ago. The legumes were collected from pits and other domestic contexts dated to the Early Pre-Pottery Neolithic B. The legumes identified include *Vicia faba* L. (faba bean), *V*. *ervilia* (bitter vetch), *V*. *narbonensis* (narbon vetch), *Lens* sp. (lentil), *Pisum* sp. (pea), *Lathyrus inconspicuus* (inconspicuous pea) and *L*. *hirosolymitanus* (jerusalem vetchling). Comparison with coeval sites in the region show how the presence of peas, narbon vetches, inconspicuous peas, jerusalem vetchlings and bitter vetches together with faba bean and lentils is unique to the Pre-Pottery Neolithic, and might indicate specific patterns in farming or storing at the onset of agriculture.

## Introduction

Agriculture is a key factor that reinforced sedentism. The ability to produce and store food-surplus drastically reduced the risk of famine and the sensitivity to environmental conditions, ultimately allowing the human population to grow and settle down. Recent archaeobotanical discoveries of early-domesticated cereals in the Fertile Crescent show that this region is rich with examples of early forms of agriculture [1]. Several notable examples of cultivation and domestication of legumes such as faba bean and chickpea have been discovered in the Levant [2–5].

Here we present new finds of legumes at the prehistoric site of Ahihud. The context of the discovery and the volume of recovered material provide insight into the early farming of staple crops and the use of other legumes for their potential value as food for humans and animals. Large amounts of *Vicia faba* L., *Lens* sp., *Pisum* sp., were found in the site of Ahihud, in the Lower Galilee in Israel. The site, which extends over 3,000 square meters, includes well-preserved remains of a Pre-Pottery Neolithic B village and an Early Chalcolithic village (Wadi Rabah culture). The remains of the legumes were collected from plastered floors, and pits in a style commonly appearing in the early phase of the Pre-Pottery Neolithic B (EPPNB) (11^th^millennium cal BP). Radiocarbon measurements of the legumes dated the findings between the ~10.250 and 9.900 cal BP. While findings of lentil and pea are quite common in the Levant in earlier phases, remains of *Vicia faba* L. are rare, and mostly found in the southern Levant [6]. Recently, wild specimens of faba beans have been discovered in the Epipalaeolithic campsite el-Wad and dated to 14.000 year ago [7]. Starting from the Pre-Pottery Neolithic the frequency of findings of faba bean begins to increase; besides the findings of Ahihud, large quantities of faba bean were discovered in storage facilities in the Middle Pre-Pottery Neolithic B Yiftah’el [4]. Smaller quantities were recovered from other Neolithic sites in the Western Asia, such as Nahal Zippori 3 [8], Horvat Galili [9], Nahal Zehora [10], Shar’Hagolan [11], Iraq ed-Dubb [12], Ain Ghazal [13], Çafer Höyük [14], Nevali Çori [15], Abu Hureyra [16], Dja’de [17] Tell el Kerkh [5] and Tell Qarassa [18].

## Context of study

### Geographical setting

**Fig 1:**
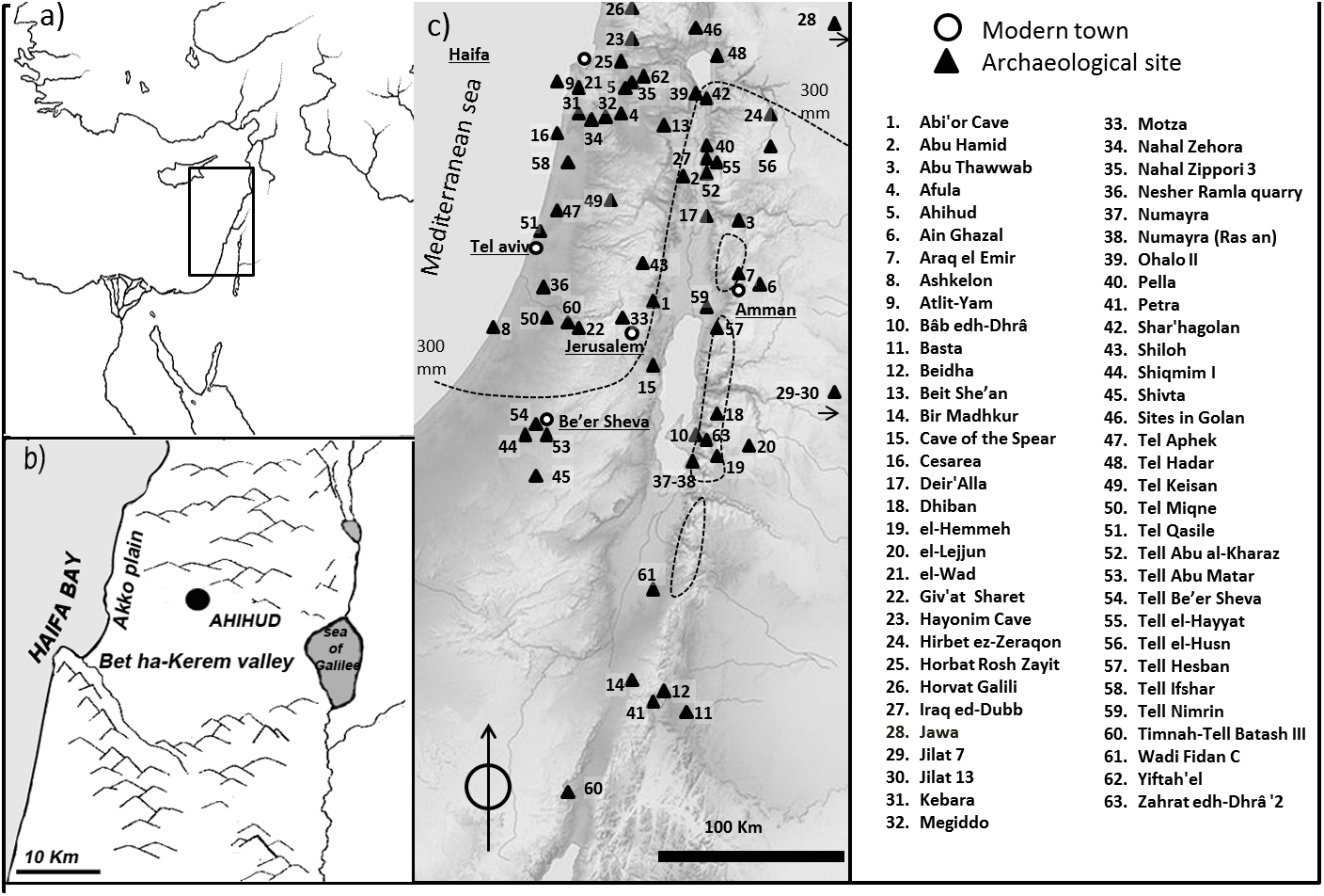
The context of study and the sites mentioned in the texts. (a) The regional setting. (b) the location of the site of Ahihud. (c) Archaeological sites in the southern Levant where remains of legumes were found. For details on the type of findings and the chronology of the sites, see paragraph ‘Legumes in the southern Levant: the archaeobotanical findings’. The dotted line represent the 300 mm of annual rainfall threshold.

The site of Ahihud is located in the Bet ha-Kerem valley in the Lower Galilee, 8 km east of the Mediterranean coast, at an altitude of 30 m a.s.l. The site is bordered to the west by the Akko coastal plain and to the east by the Har Gilon ridges (340 ma.s.l.) (**Fig 1**). Terra rossa, a clay-rich sediment, is the most common soil type of the Bet ha-Kerem valley and the Akko plain, while the western margin of the Har Gilon ridges are characterized by limestones formations [19].

The area around Ahihud is characterized by a Mediterranean warm-temperate climate with hot and dry summers and moist, cool winters. Temperatures range from 17 to 31°C, and rainfall is around 550 mm p.a, with the rainy season spanning from October to May [20]. Several biomes can be observed in Lower Galilee, according to Danin and Orshan [21] *Quercus ithaburensis* forests cover the slopes at an altitude between 0 and 500 m a.s.l. Typical arboreal components of this kind of forest are *Styrax officinalis*, *Pistacia atlantica*, *P*. *palaestina*, *Quercus calliprinos*, shrubs like *Majorana syriaca* and many herbaceous plants, e.g. grasses. Synanthrophic vegetation, within intensely cultivated areas, can be found on the Akko coastal plain.

### Archaeological site

In 2012/2013 a salvage excavation was carried out on the northern slope of a chalk hill at the northeastern end of the Akko Plain. The hill, which was cut when Highway 85 was initially paved, is situated in the Nahal Hilazon flood plain. The excavation of 1,800 square meters exposed two settlements: a Pre-Pottery Neolithic B village, located right on top of the bedrock, on the east, and an Early Chalcolithic village (Wadi Rabah culture), on the west of the excavated area.

The PPNB occupation of Ahihud was defined based on the lithic tools found at the site. The assemblage included several dozen arrowheads, mostly Helwan and el Khiam points, which enabled the dating of the layers to the early phase of the Pre-Pottery Neolithic (EPPNB) [22]. The tool assemblage also includes tranchet axes and sickle blades, commonly used for domestic tasks. Obsidian artifacts were found, and since obsidian is not locally available, its presence in the site proves that the occupants of Ahihud had access to tools made of raw material coming from the northern Levant. So far, only a few PPNB settlements in the Galilee and central Israel have an Early phase (EPPNB) and those are Horvat Galil (Upper Gallilee) [23] and Motza (Judean hills) [24, 25].

**Fig 2:**
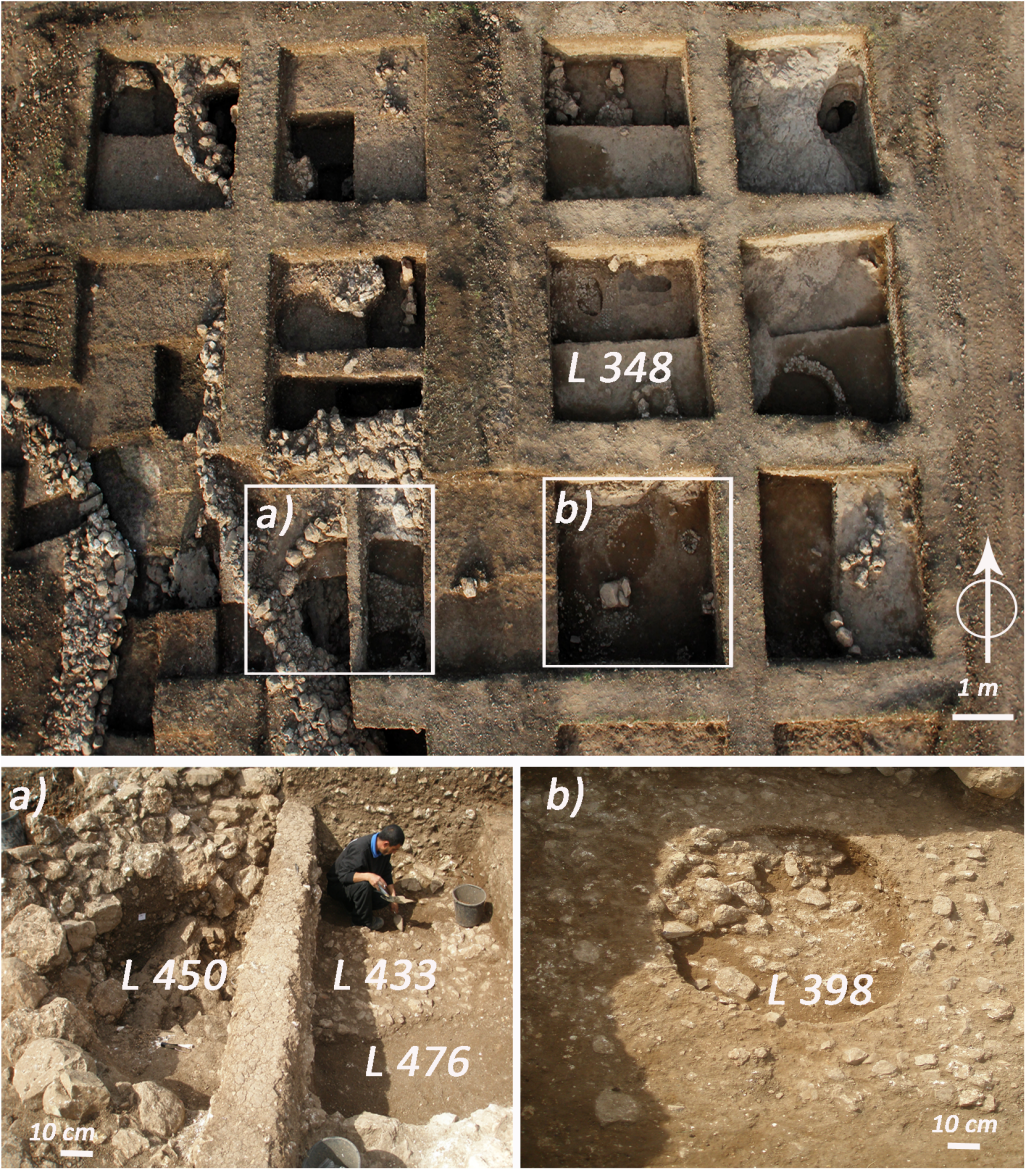
The EPPNB area in Ahihud with the distribution of the most important Loci. (a) a detail of the silo L 450 and the floors L 433 and L 476. (b) the storage pit L 398. The individual in this figure has given written informed consent (as outlined in PLOS consent form) to publish these case details.

The excavation of the EPPNB phase revealed several domestic units, such as those found in square D14, where a floor (Locus) L 348 made of small stones and basalt grinding stones was discovered. At least two of the domestic units of the EPPNB phase included pits. The first, L 450, was a large and deep pit (ca. 2m in diameter and 1,4m in depth), that was partially cut into the bedrock and had a thick constructed stone wall that defined the upper surface. In L 450 the seeds were found mostly in the bottom, about 70 cm below the entrance of the silo. The second pit, L 398, was much smaller and shallower (ca 60 cm in diameter and max 30 cm deep), and was dug in the floor of a dwelling (**Fig 2**). According to the stratigraphy, L 450 was already concealed when L 398 was in use, and should therefore be slightly older than L 398 [22].

The other contexts from which the seeds were recovered are floors of dwelling where EPPNB artifacts were found. These are L 433 and L 476 in square E14, and L 393 which is just below the above mentioned L 348 in square D14 (**Fig 2**). Few seeds were also collected from L 442, a reddish layer just above the bedrock that did not include any particular feature.

## The origin of the archaeobotanical assemblage—approaches and perspectives

Several studies have shown that many factors such as agronomic practices, cultural choices and natural factors, influence the state of the archaobotanical remains [26]. Among those factors, crop-processing is one of the most relevant because it acts as a filter that separates crops from unwanted plants depending on their size and weight [27].

Typically, observations of the botanical composition of archaeological samples are combined with information inferred from ethnographic models [26–28]. Parameters such as density of charred seeds in the sediment, state of preservation of the seeds and degree of distortion are used to distinguish between materials burnt *in situ* and refuse discarded in secondary or tertiary contexts [29]. The archaeological context can be used to assess the original function and possible re-functionalization of storage and to evaluate the impact of post-depositional events [30].

Finally, the study of microarchaeological evidence, such as phytoliths and sediments, has proven to be extremely helpful to study the processes that lead to the formation of refuse deposits and storages [31]. Once the information on the archaeological and archaeobotanical records are available, the comparison with ethnographic models can help to shed light on the origin of the macroremains and to understand the processes involved in the formation of the ancient deposits.

The presence of a two pits full of legumes in Ahihud offers profound insight into the origin of farming and storage of legumes at the onset of agriculture. In order to exploit the potential of such discovery, the study of the macroremains was combined with the radiocarbon dating of the seeds. Additionally, Fourier-Transform Infrared Spectroscopy (FTIR) was used to analyze the sediments in which the seeds were buried and to look for traces of *in situ* combustion. Finally, the information inferred from the archaeological record was compared to the ethnographic models to distinguish between product and by-products of crop processing, storage products and discarded refuses.

## Material and methods

The Israel Antiquities Authority granted the permission to excavate Ahihud and work on the material collected during the fieldwork. Authorization number 6663.

### Fourier transform infrared spectrometry (FTIR)

Samples of sediment collected in the pits (L 450, 398) and on the floors were analyzed using FTIR to look for the presence of ash and of any other sign of fire activity in the clay, following the model proposed by Berna [32].

A few tens of micrograms of sample were ground using an agate mortar and pestle. About 0.1 mg of the sample was mixed with about 80 mg of KBr (IR-grade). A 7 mm pellet was made using a hydraulic press (Specac). The spectra were collected between 4000 and 500 cm^-1^ at 4 cm^-1^ using a c Nicolet 380 instrument (Thermo) and OMNIC v.9 software.

### Archeobotany

Samples for archaeobotanical analyses were collected in Ahihud during the excavation in 2013, from 11 loci, for a total of 27 baskets. The majority of the baskets come from the storage pits L 450 and L 398. A total of 140 liters of sediment, coming from 9 baskets collected in L 450, 476 and 398, was sieved using sieves of mesh 3 mm and 1 mm. The residue of the sieving was sorted under a magnification lamp and the plant material was separated from flint fragments and other stones, and stored in glass vials. An additional 18 baskets contained vegetal material, which had been hand-picked during the excavations from Loci 348, 393, 433, 442 and Loci 450, 476 and 398. Seed morphologies were analyzed under a binocular microscope (Leica M80), and measurements of seed size (i.e. seed length, breadth, thickness and diameter, hilum and radicle length) were made using an imaging analysis program (LAS V 3.8).

The archaeobotanical material was sorted and dived into groups according to the seed size and the seed outline. The criteria used for the identification to the species level of each group include qualitative parameters and quantitative measures of anatomical features. The qualitative parameters are: seed outline, which can be pyriform, sub-triangular, oblong, sub-lenticular, sub-spherical; sub-rectangular, and hilum outline, which can be circum-linear, linear, oblong, wedge, oval [33–35]. The quantitative parameters are: seed circumference, absolute hilum length, relative hilum length (as % of the seed circumference), absolute radicle length and relative radicle length (as % of the seeds circumference). The absolute hilum length, relative hilum length and hilum outline were inferred from the hilum fingerprint on the cotyledons. The parameters where chosen according to the classification of modern legumes proposed by Gunn [33], Perrino *et al*. [34] and Chernoff *et al*. [35].

Photographs of the seeds were taken using a LEO Supra 55VP scanning electron microscope (SEM) with the secondary electron detector.

The reference collection of local plants, provided by the Volcani Center-Israel and the Leibniz Institute of Plant Genetics and Crop Plant Research (IPK)-Germany, was used for the identification of the plant remains. In addition, atlases of plant anatomy were used to refine the identification [36, 37].

### Radiocarbon dating

Thirteen samples were selected for radiocarbon dating. Six were *Vicia faba* L., and the rest were single specimens of *Lathyrus hierosolymitanus*, *L*. *incospicuus*, *Lens* sp., *Pisum* sp., *Vicia ervilia*, *V*. *narbonensis* and *Triticum turgidum* ssp. *dicoccum/dicoccoides*. The samples were selected from the plant remains found L 348, 398, 433, 442, 476 and 450.The samples were pre-treated, graphitized and measured by the Accelerator-Mass Spectrometer at the D-REAMS Radiocarbon Laboratory of the Weizmann Institute of Science, Israel. The legumes (~30 mg of material) were cleaned using Acid-Base-Acid treatment as in Yitzhaq [25].The samples prepared for dating were combusted to CO_2_ in quartz tubes containing about 200 mg of copper oxide (Merck) and heated to 900°C for 200 min. The CO_2_was divided into 3 aliquots and each was reduced to graphite using cobalt (Fluka) (about 1 mg) as a catalyst and hydrogen at 700°C for 20 hr. The ^14^C ages were calibrated to calendar years BP and BC using the r.5 IntCal13 atmospheric curve [38] using the software OxCal v 4.2.4 [39].

## Results

### Fourier transform infrared spectrometry (FTIR)

**Fig 3:**
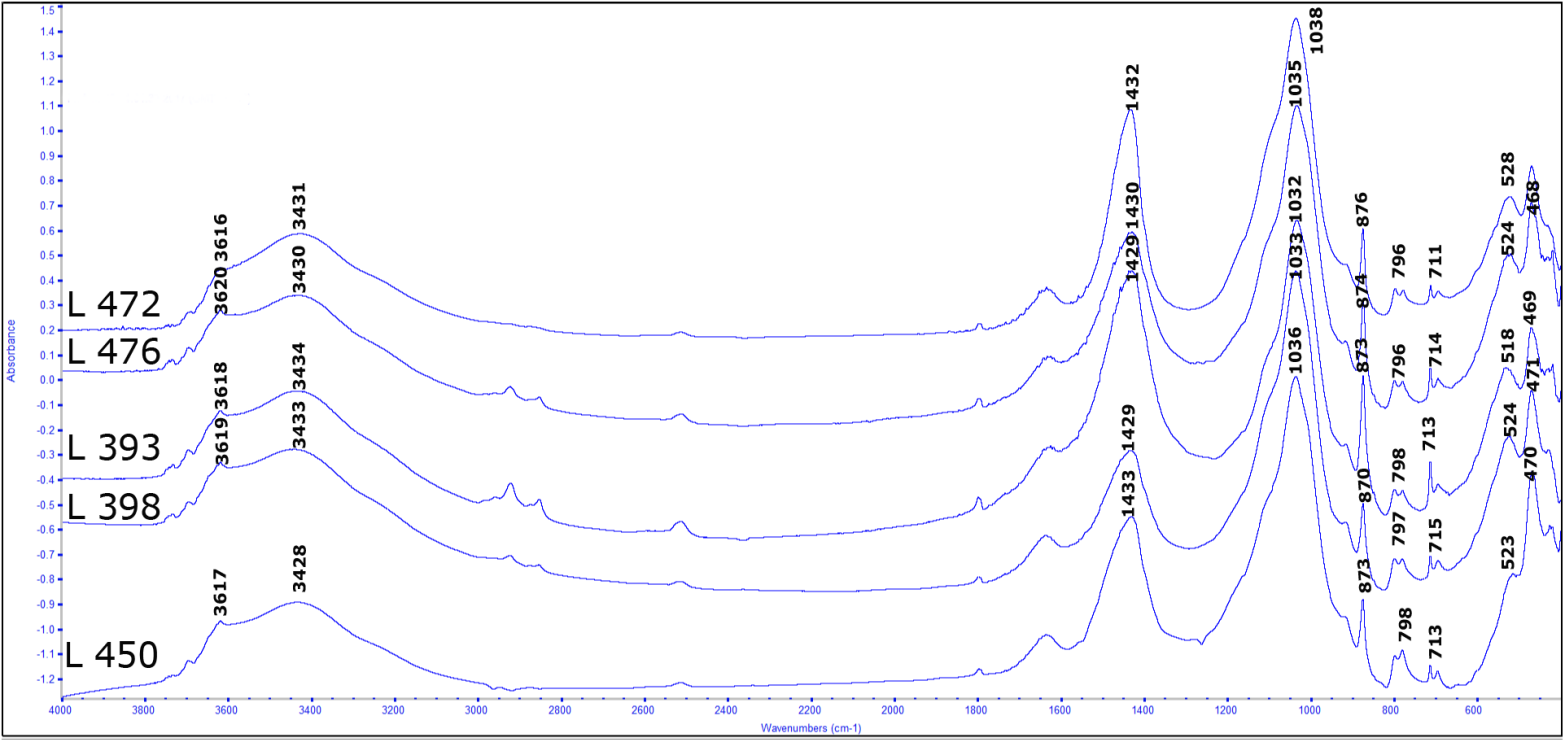
Infrared spectra of sediments from different locations at Ahihud. All the spectra show typical peaks of unaltered clay, such as those of the structural water (3695 and 3620 cm^-1^) and the peak at 1032 cm^-1^.

The spectra obtained from the different contexts show remarkable similarities (see **Fig 3**). The local clay minerals, mostly of montmorillonite type, are characterized by peaks at 3695 and 3620 cm^-1^, which are indicative of the clay structural water, while the peaks at 1032, 915, 798, 778, 535 and 470 cm^-1^ are indicative of the silica and alumina components of clay.

The peaks at 1430, 877, 713 cm^-1^ are characteristic of calcite, which could be geogenic (chalk of the original matrix of the sediment) or pyrogenic (ash). To identify the type of calcite present in the sediment, we used the model proposed by Regev *et al*. [40] and we plotted the normalized heights of the peak v^2^ (877 cm^-1^), versus the v^4^ (713 cm^-1^). The results showed that the calcite present in the sediment in not pyrogenic. The absence of ash was an indirect indication that the sediment contained few phytoliths. The phytoliths were not expected in the pits because legume seeds have few or no phytoliths [41]. The absence of peaks characteristic of opal, such as the peak at 1100 cm^-1^, was a further proof that phytoliths were scarce in the sediment.

The FTIR analysis was also used to detect sign of exposure to fire in the clay, provided that the fire has had reached temperature above 400°C. Upon exposure to high temperature, clay minerals are known to transform in the following way: at 400–500°C, the structural water is evaporated, so the peaks at 3695 and 3620 cm^-1^ disappear. Between 500 and 700°C, several peaks either disappear (915–535 cm^-1^) or shift to higher wavenumbers (1032 shift to 1048 cm^-1^). Above 800°C, the main absorption bands shift progressively to wavenumbers higher than 1080 cm^-1^ [32]. In our sediment, none of these signs was present; indeed, the structural water was present in samples from both the floors and the pits.

The FTIR analysis of the samples from Ahihud showed that the samples from the floors and the pits were equally depleted of phytolith (and ash). FTIR analysis confirms that the temperature reached during the fire never exceeded 200–300°C. Experimental burning of legumes demonstrates their tendency to explode when burnt above 200°C [3]. The state of preservation of the legumes provides additional evidence that the fire responsible for charring the legumes never exceeded 200°C.

### The archaeobotanical assemblage

In total, 3052 whole seeds and 4162 fragments were recovered from the EPPNB contexts (**Table 1**). Given the meshes used for the sieving, it is possible that some weeds shorter than a millimeter escaped the sorting.

**Table 1:** List of identified seeds from Ahihud.

Square	Locus	Basket	Seeds												
			*Triticum* *turgidum* ssp. *dicoccum* */dicoccoides*	Leguminosae unidentified	*Astragalus* sp.	*Lathyrus hierosolymitanus*	*Lathyrus inconspicuus*	*Lens* sp.	*Pisum* sp.	*Vicia ervilia*	*Vicia narbonensis*	*Vicia* sp. (small type)	*Vicia faba* (number of fragments)	Total seeds	Volume (Lt)
**D14**	**348**	**1867**	-	-	-	-	-	-	-	-	-	-	1	1	/
	**393**	**1919**	-	-	-	-	-	-	-	-	-	-	2 (3)	2	/
**D13**	**398**	**1937**	-	-	1	10	50	7	13	14	-	11	100 (80)	206	/
		**1922**	-	10	-	1	5	4	46	-	-	10	85 (38)	161	/
		**1928**	-	-	-	-	-	-	-	-	-	-	64 (120)	64	/
		**General**	-	150		25	138	195	272	22	7	40	161 (356)	1010	50
**E14**	**442**	**2803**	-	-	-	-	-	-	-	-	-	-	23 (50)	23	/
	**433**	**2644**	-	-	-	-	-	-	-	-	-	-	3 (1)	3	/
		**2674**	-	-	-	-	-	-	-	-	-	-	20 (179)	20	/
		**2684**	-	-	-	-	-	-	-	-	-	-	4 (9)	4	/
	**450**	**2657**	-	-	-	-	-	-	-	-	-	-	170 (90)	170	20
		**2714**	-	-	-	-	-	-	-	-	-	-	75 (635)	75	10
		**2715**	-	-	-	-	-	-	-	-	-	-	384 (445)	384	/
		**2722**	-	-	-	-	-	-	-	-	-	-	55 (253)	55	10
		**2729**	-	-	-	-	-	-	-	-	-	-	301 (443)	301	10
		**2735**	-	-	-	-	-	2	-	-	-	-	103 (636)	105	10
		**2740**	-	-	-	-	-	-	-	-	-	-	324 (647)	324	10
		**2746**	-	-	-	-	-	-	-	-	-	-	17	17	/
		**2797**	-	-	-	-	-	2	-	-	-	-	4 (33)	6	/
		**General**	-	-	-	-	-	-	-	-	-	-	66 (54)	66	10
		**2686**	-	-	-	-	-	-	-	-	-	-	24	24	/
	**476**	**2795**	-	-	-	-	-	-	-	-	-	-	19 (18)	19	10
		**2797**	4	-	-	-	-	-	-	-	-	-	9	13	/
		**2805**	-	-	-	-	-	-	-	-	-	-	29 (62)	29	/
**Total**			**4**	**160**	**1**	**36**	**193**	**208**	**316**	**36**	**7**	**61**	**2053 (4162)**	**3052**	**140**
**%**			**0,3**	**5**	**0,2**	**1**	**6**	**7**	**11**	**1**	**0,5**	**2**	**66**	**100**	

Information about the contexts (square, locus, basket) are provided on the first three colums on the left, while the volume of the sieved sediment is provided on the last colum in the right. (-) indicates that no seeds of that species were found. (/) indicates that material from that basket was handpicked during the excavation. The fragments of *Vicia faba* are reported in brachets.

The gross of the remains come from two pits, L 450 and 398 while fewer remains come from the other Loci. The sampling strategies might account for the differences in the number of remains and species found in the pits *vs* the floors (see paragraph on material and methods).

Except for a few caryopses of *Triticum turgidum* ssp. *dicoccum/dicoccoides* (emmer) found in L 476, the rest of the assemblage is composed of different type of legumes (**Table 1**). Eight major groups were identified using the following parameters: seed outline (*so*), seed circumference (*c*), hilum outline (*hs*), absolute hilum length (*h*), relative hilum length (*h % c*), absolute radicle length (*r*), relative radicle length (*r % c*).

**Fig 4:**
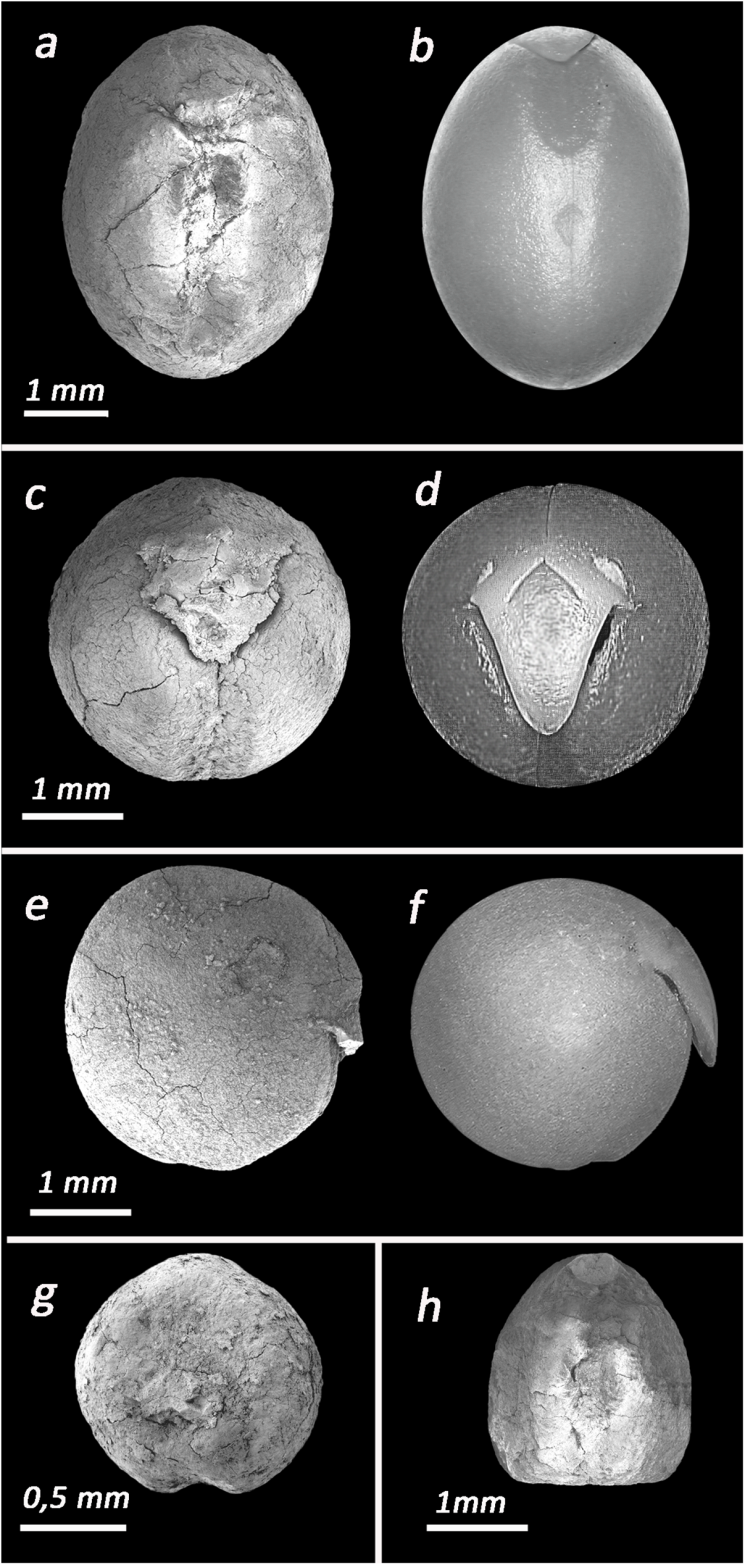
Photographs of the most common taxa in Ahihud and modern comparisons. (a) *Lathyrus inconspicuus* (Ahihud); (b) *Lathyrus inconspicuus* (modern); (c) *Pisum* sp. (Ahihud); (d) *Pisum elatius* (modern); (e) *Lens* sp. (Ahihud); (f) *Lens orientalis* (modern); (g) *Lathyrus hierosolymitanus* (Ahihud); (h) *Vicia* sp. (small type) (Ahihud).

Group 1- *Pisum* sp. -*so*: sub-spherical, *c*: 8.5±0.3 mm, *ho*: oval, *h*: 1±0.1 mm, *h % c*: 11%, *r*: 1±0.1 mm, *r % c*: 11%. At the present, three wild species of *Pisum* grow in the Lower Galilee: *P*. *fulvum*, *eleatius* and *P*. *sativum* subsp. *humile* [42]. Based on the sole morphometry of the archaeobotanical remains is not possible to reach an identification to the species level (**Fig 4C**).

Group 2- *Lens* sp. -*so*: sub-lenticular, *c*: 8.2 ±0.3 mm, *ho*: linear, *h*: 0.9±0.1 mm, *h % c*: 12%, *r*: 1±0.1mm, *r % c*: 12%. Three species of lentils grow in the region under study, the wild *Lens orientalis* and cultivated *L*. *ervoides* and *L*. *culinaris* [42]. As for the *Pisum*, it is not possible to identify the archaeobotanical remains to the species level (**Fig 4E**).

**Fig 5:**
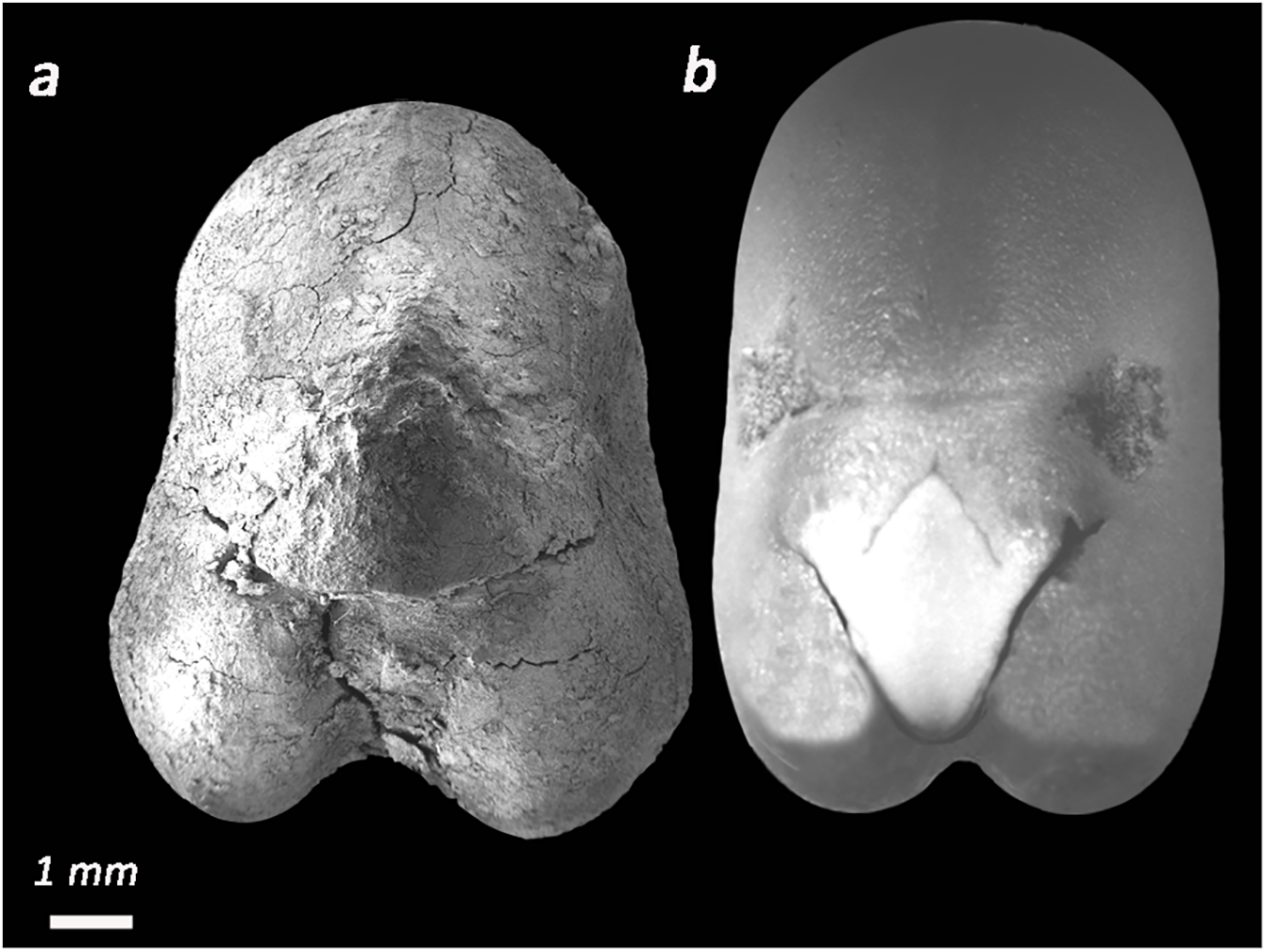
*Vicia faba*. (a) specimen from L 450; (b) modern faba bean.

Group 3- *Vicia faba*- *so*: pyriform, *c*: 17.6 ±1.3 mm, *ho*: linear, *h*: 3±0.2 mm, *h % c*: 18%, *r*: 3.5±0.5 mm, *r % c*: 20%. Compare to other local species of *Vicia* of large size, such as V. *galeata*, V. *galilea* and V. *narbonensis* susbp. *aegyptiaca*, the remains presents a unique pyriform outline, strongly compressed, with a very long hilum, that covers about 1/5 of the total circumference, and a long radicle, which is located along the vertical axes of the seeds, at the junction of the two cotyledons. These characteristics, which are typical of faba beans and cannot be found in other vetches, allow a secure identification of the seeds (**Fig 5**).

**Fig 6:**
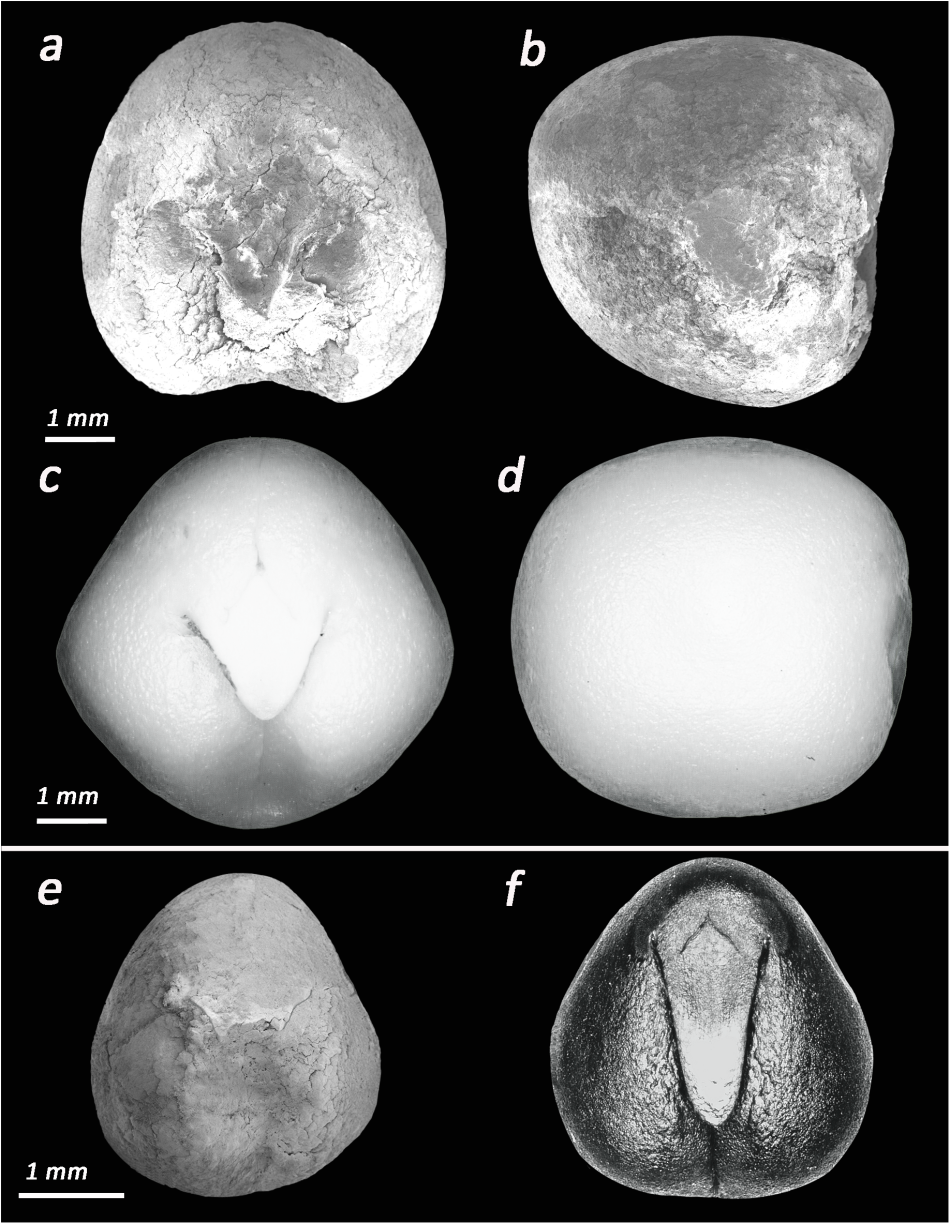
Archeological and modern Vicieae. (a-b) ventral and lateral views of *Vicia narbonensis* (Ahihud); (c-d) ventral and lateral views of *Vicia narbonensis* (modern); (e) *Vicia ervilia* (Ahihud); (f) *Vicia ervilia* (charred modern).

Group 4-*Vicia ervilia* -*so*: sub-triangular, *c*: 9±0.2 mm, *h*: 1.2±0.5mm, *h % c*: 13%, *r*: 1.8±0.1 mm, *r % c*: 20%. While the majority of medium size vetches are spherical or rounded with relatively short radicle, the seeds of this group present sub-triangular shape and a long radicle that allow to attribute the seeds of Group 4 to the species *V*. *ervilia* (**Fig 6E**).

Group 5- *Vicia narbonensis*- *so*: sub-rectangular, *c*: 14±1.1 mm, *ho*: oval/wedge, *h*: 1.5±0.3 mm, *h % c*: 11%, *r*: 1.1±0.2 mm, *r % c*: 8%.The group of vetches of medium-large size with a sub-rectangular seed outline and short radicle includes *V*. *palaestina* and *V*. *peregrina* and *V*. *narbonensis*. Among these, only *V*. *narbonensis* presents a wedge hilum that resembles that of the legumes of Group 5 (**Fig 6A and 6B**).

Group 6- *Lathyrus hierosolymitanus—so*: sub-rectangular, *c*: 12±1.5 mm, *ho*: linear *h*: 1.1±0.1mm, *h % c*: 9%, *r*: 2±0.1mm, *r % c*: 17%. *Lathyrus hirosolymitanus* and *L*. *gorgoni* are, among the medium-size vetchlings, the only ones that present a sub-rectangular outline. They can be distinguished on the base of the outline and relative position of the hilum. In the case of *L*. *gorgoni* the hilum is oval and spreads to the base of the radicle. *L*. *hirosolymitanus* has a linear hilum that is clearly distinct from the radicle; similar features are found in legumes of Group 6 (**Fig 4G**).

Group 7: *Lathyrus inconspicuus–so*: oblong/sub-spherical, *c*: 9±0.2 mm, *ho*: oval, *h* 0.5±0.1 mm, *h % c*: 5%, *r*: 0.7±0.1 mm, *r % c*: 8%. Seeds that belong to the species *Lathyrus inconspicuous* are medium-small size legumes that have unique oblong/sub-spherical outline and a very small radicle and oval hilum. The presence of these very characteristics on the legumes of Group 7 allow identifying them as *L*. *inconspicuus* (**Fig 4A**).

The two pits, which have the most abundant assemblage, differ in the number of specie; L 450 contains almost exclusively faba beans, while L 398 also contains *Astragalus* sp., *Lens* sp., *Vicia ervilia*, *V*. *narbonensis*, *Lathyrus incospicuus* and *L*. *hierosolymitanus*. Faba bean represents 66% of the total seeds collected in the site, followed by pea, bitter vetch and inconspicuous pea, which represents respectively 11%, 7% and 6% of the total archaeobotanical assemblage (**Table 1**).

### Size analysis and harvest homogeneity

The large number of pulses found at Ahihud offer an unprecedented opportunity to study the biometric characteristics of ancient faba bean, pea and lentils in-depth at the early stage of legume farming. The biometric parameters (e.g. length, breadth, etc.) of the seeds were measured to evaluate the heterogeneity in the size of the harvested legumes.

In total, 462 seeds were measured, 270 were faba beans collected from the pits L 450 and 398, and from floors L 433, 476,442. The difference in the number of specimens measured for each context also depends on the number of faba beans available and the state of preservation, which was better for seeds from pits than from those collected from the floors. In addition to faba beans, 96 pea, and 96 lentils from L 398 were measured (**Table 2**).

**Table 2:** Results of the biometric analysis of *Vicia faba*, *Lens* sp. and *Pisum* sp.

(mm)	L 450			L 433			L 476			L 442			L 398				
	*Vicia faba*															*Pisum* sp.	*Lens* sp.
	L	B	T	L	B	T	L	B	T	L	B	T	L	B	T	D	D
**Average**	6.1	4.9	4.5	6.9	4.8	4.5	6.5	5.3	5.0	6.4	5.2	4.9	5.9	4.7	4.2	2.7	2.6
**St Dev (+/-)**	0.5	0.4	0.4	0.7	0.6	0.6	0.6	0.5	0.5	0.7	0.6	0.5	0.6	0.5	0.5	0.3	0.2
**Maximum**	7.2	6.1	5.6	7.3	5.9	5.5	7.3	6.1	6.7	7.9	6.4	5.7	7.3	6.0	5.5	3.6	3.5
**Minimum**	4.4	3.6	3.5	5.1	4.2	3.7	5.3	4.0	4.0	5.2	4.1	3.5	4.8	3.8	2.2	1.9	2.0
**Number of specimens**	128			12			35			18			77			96	96

L = Length, B = Breadth; T = Thickness; D = Diameter. The last row provides the total number of seeds measured.

Biometric parameters of legumes are sensitive to the charring temperature. In order for the legumes to maintain their characteristic shape, they must be charred at temperatures between 200°C and 230°C [43]; above this threshold, the seeds are likely to crack and lose their original shape [44]. Since our samples were not cracked and thus in a good state of preservation, they were likely charred at low temperatures (~200°C) and as a consequence the size was homogenously reduced [3].

The average lengths, breadths and thicknesses of the archeological faba beans are shown in **Table 2**. The size of the seeds coming from the same Loci is quite homogenous, and the standard deviation of length, thickness and breadth is around ~10%. Seeds coming from floors L 433, L 476, and from the layer L 442 are larger and longer than those coming from storage pits L 450 and 398, as shown in (**Table 2**). Although the faba beans stored in L 450 and L 398 have similar size, we can exclude that they come from the same harvest, since the two pits were use in different periods, as shown by stratigraphical observation and radiocarbon dating. The relative homogeneity in the size of the stored faba beans might, instead, point out at a specific variety of such legume, which was used by the local farmers over a relatively short period.

**Fig 7:**
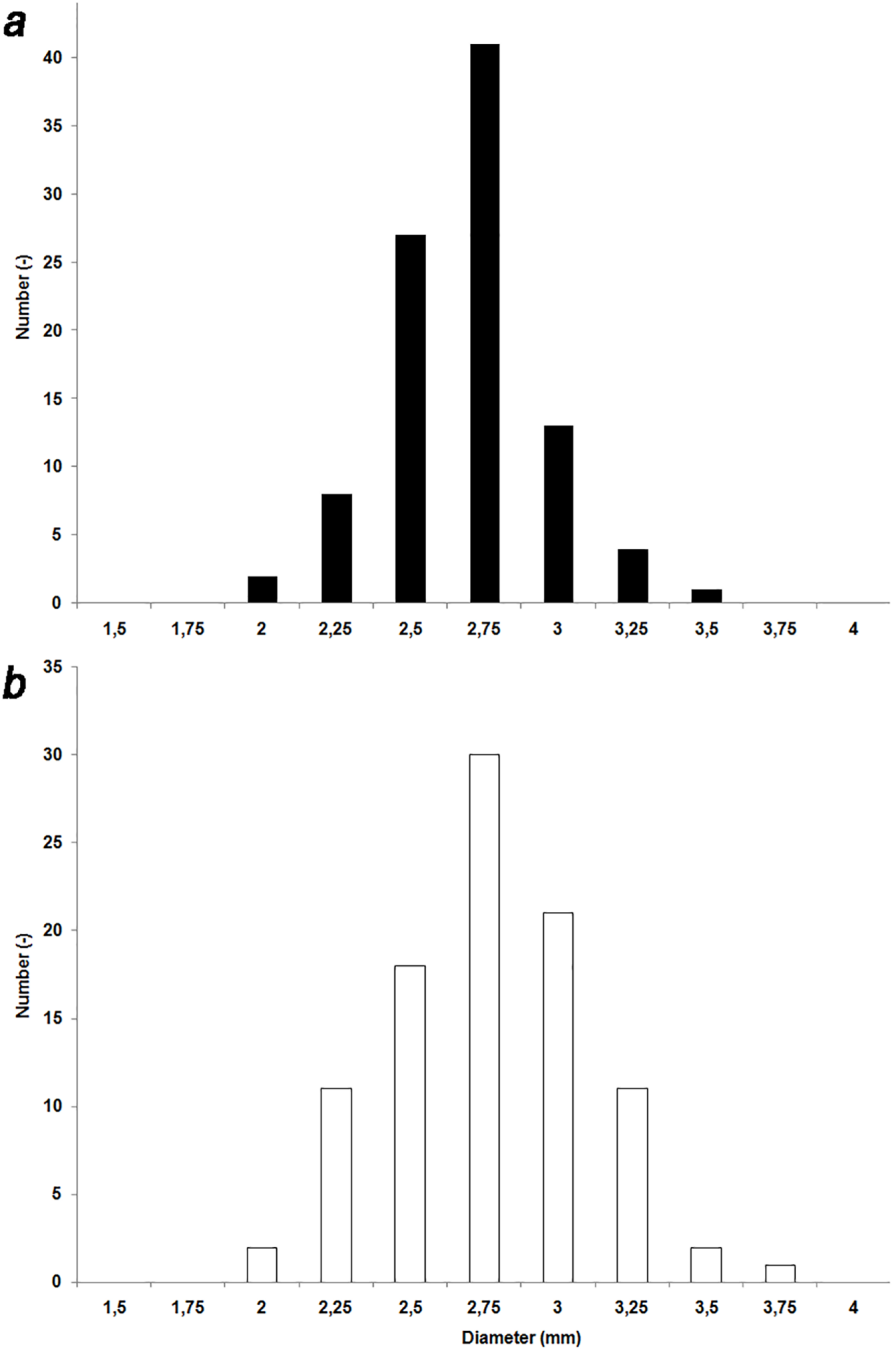
**Histograms plot showing the distribution of the size of (a) 96 seeds of *Lens* sp.; (b) 96 seeds of *Pisum* sp.** Each bar represents the frequency of diameters occurring at interval of 0.25 mm.

The analysis of lentils and peas collected in L 398 show that seeds have diameters mostly comprised between 2.5 and 3 mm, with a peak at 2.75 mm (**Table 2** and **Fig 7**). The standard deviation of lentils and peas is around ~10% (respectively 0.2 and 0.3 mm); thus size of the lentils and peas is homogenous.

### Chronology

**Fig 8:**
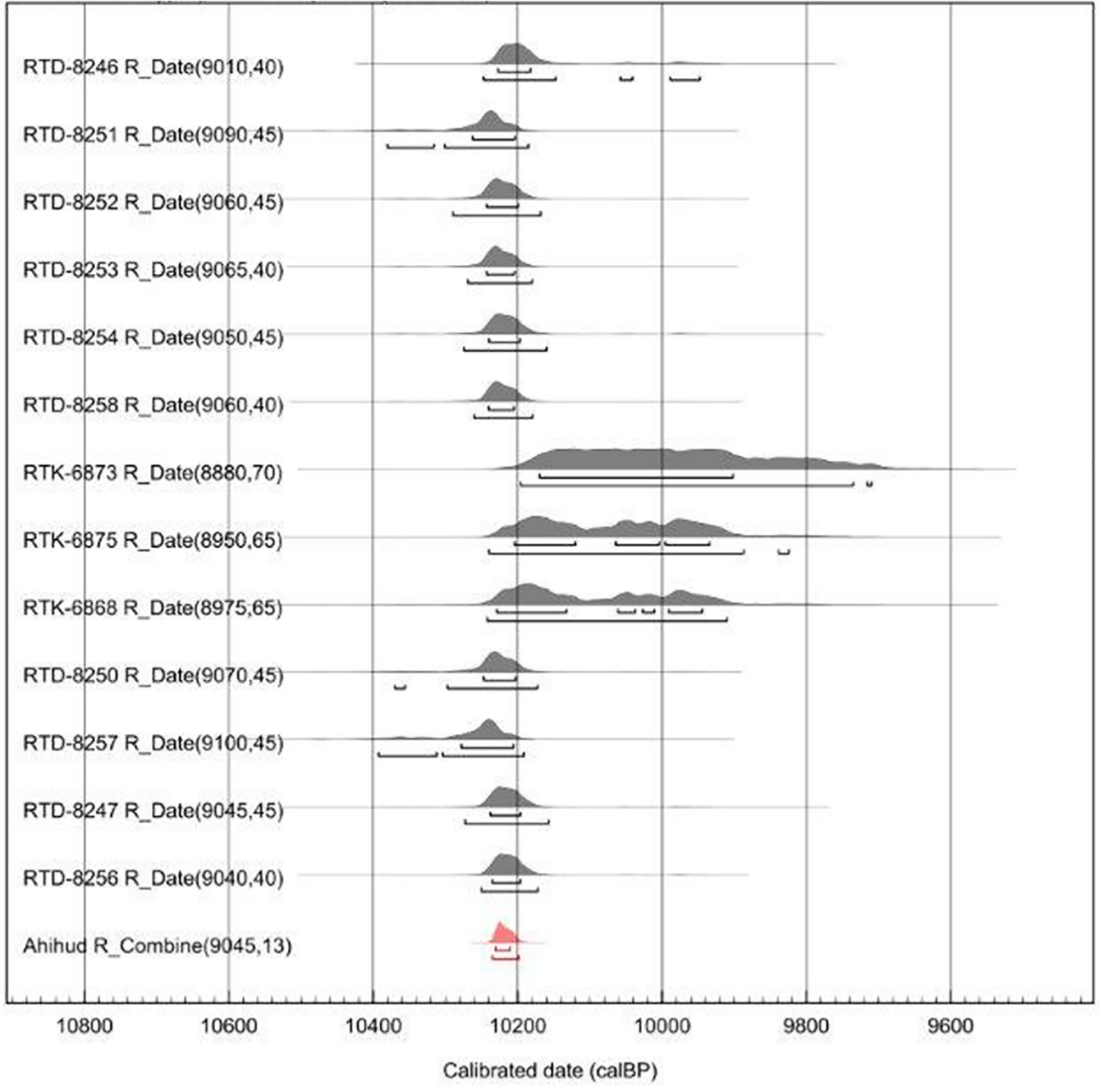
Curves of the calibrated dates. The ^14^C ages were calibrated to calendar years BP using the r.5 IntCal13 atmospheric curve [38] using the software OxCal v 4.2.4 [39].

The radiocarbon dating obtained from the thirteen samples (**see Table 3**) cover a short range of time, namely~200/300 years, between ~10.250 and 9.900 cal BP (68.2%). The oldest date comes from the storage pit L 450 (10.280–10.210 cal BP; RTD-8257), while the youngest date was obtained from the storage pit L 398 (10.170–9.900; RTK-6873) (**Fig 8**). When the dates are combined using OxCal, the time of occupation of the site is reduced to less than 50 years (see **Table 3**). Overall, the radiocarbon chronology match with the latest part of the chronological range proposed for the Early Pre-Pottery Neolithic B [23].

**Table 3:** Radiocarbon dating.

Lab ID	Locus	Type of remain	Uncal BP	Cal BP 1σ (68.2%)	Cal BP 2σ 94.5%
RTD-8246	348	*Vicia faba*	9.010±40	10.230–10.185	10.250–9.950
RTD-8251	398	*Lathyrus hierosolymitanus*	9.090±45	10.265–10.205	10.380–10.185
RTD-8252		*Lens* sp.	9.060±45	10.245–10.200	10.290–10.170
RTD-8253		*Lathyrus incospicuus*	9.065±40	10.245–10.205	10.270–10.180
RTD-8254		*Vicia ervilia*	9.050±45	10.240–10.200	10.275–10.160
RTD-8258		*Vicia narbonensis*	9.060±40	10.240–10.205	10.260–10.180
RTK-6873		*Pisum* sp.	8.880±70	10.170–9.900	10.200–9.710
RTK-6875		*Vicia faba*	8.950±65	10.205–9.935	10.240–9.825
RTK-6868	433	*Vicia faba*	8.975±65	10.230–9.945	10.235–9.920
RTD-8250	442	*Vicia faba*	9.070±45	10.250–10.205	10.370–10.175
RTD-8257	450	*Vicia faba*	9.100±45	10.280–10.210	10.395–10.190
RTD-8247	476	*Vicia faba*	9.045±45	10.240–10.200	10.275–10.160
RTD-8256		*Triticum turgidum* ssp.*dicoccum*	9.040±40	10.235–10.200	10.250–10.170
Ahihud Combined			9.040±13	10.230–10.210	10.240–10.200

The table includes the tracking number provided by the laboratory (Lab ID), the details of the context of finding, the species and the ^14^C age associated to each sample (uncal BP). The ^14^C ages were calibrated to calendar years BP and BC and the results are reported for 1σ range of probability, which corresponds to 68.2%.

## Discussion

### Wild or farmed legumes?

The distinction between wild and domesticated forms of plants is mostly based on the development of certain mutations that are beneficial to human exploitation; in a few cases, trace of the mutations can be identified in the archaeobotanical remains (i.e. non-shattering ears of cereals) [45].

The distinction of wild and domestic forms in legumes is more complicated, because the main mutations associated with domestication are reduced seed-dormancy and the loss of dispersal mechanisms (indehiscent pods) which do not leave visible trace on the seeds [46]. Other mutations, such as reduce seed-coat thicknesses [47] and increased seed-size [48], are secondary developments selected during the process of crop improvement [5, 49, 50].

The inhibition of germination is a major factor that prevent humans from obtaining profitable yields of wild legumes. Experiments prove that the collection of wild peas, lentil and chickpea is not efficient due the low-yield potential associated to the dormancy and to the irregular distribution of the wild species in the natural habitat [51, 52].

Dormancy is a common trait of the wild progenitors of the most important legumes (i.e. *Lens culinaris* ssp. *orientalis*, *Pisum humile*, *Cicer reticulatum*, *Vigna radiata* subsp. *sublobata* and *Phaseoulus vulgaris*) so it is likely that the wild ancestor of *Vicia faba* had similar mechanisms to delay germination during unsuitable ecological conditions, when the probability of seedling survival is low. Wherever large stocks of legumes are found in archaeological sites, plants with domestic-traits (non-dormant/non-dehiscent) must have been used.

Given the large number of faba beans recovered at the site, we can assume that this plant was already domesticated, whereas it is unclear if lentil and pea were domesticated too. Evidence of the exploitation of lentil dates back to the Palaeolithic (see Kebara [53], but it is only during the PPNB that large stocks of the legume are found in several sites in the Levant and the Fertile Crescent [54]. Noticeably, million lentils and thousands faba beans were discovered in the nearby site of Yiftah’el suggesting that the domestication of lentil was already advanced in the Middle PPNB [55, 56]. The spectrum of legumes found in Ahihud is wider than that discovered in Yiftah’el, suggesting that Ahihud represents an earlier stage of legume domestication, when farmers were experimenting with different species, some of which would eventually became staple crops.

### Crop-processing and economic importance of the legumes

The large quantities of legumes found in Ahihud raise questions about the economic importance of the legumes and the processes involved in the formation of the deposits. While is far too simplistic to measure the economic importance of a plant based only on its abundance in the archaeological record, its status can be assessed by looking at the type of domestic activities to which it was subjected [29], principally because plants that are economically important were usually stored or cooked while weeds were used for roofing or fuelling or were dumped into middens [57].

Given these premises, it is therefore crucial to identify the function of the contexts where the legumes were found and the kind of processing activities with which the legume were associated.

The FTIR, which was carried out to highlight the original use of the pits L 450 and 398, proved to be less informative than expected. The analysis showed no remarkable differences in the composition of sediments coming from floors or pits, in fact the sediments were made of a similar matrix of clay and calcite and showed no sign of thermal alteration or ash. The absence of visible changes in the spectra of the sediments depended by the low temperature reached by the fire, which charred the legumes without compromising their preservation and prevented the formation of ash.

The study of the archaeological contexts was more informative and offered more insights about the possible function of the pits. Locus 450 is a large and deep pit, partially cut into the bedrock and has a thick constructed stone-wall that defines the upper surface. Considerable effort was required to cut into the bedrock and to build a stone-wall around the entrance, and therefore we can assume that the structure was constructed for a specific function.

The other pit, L 398, is shallower than L 450 and presents a simpler architecture. Its structure might recall that of common refuse pits, but similar structures are found abundantly in the nearby PPNB site of Yiftha’el, where the pits were filled with faba beans, lentils and a few grains of emmer [4; 55,56]. Based on cultural material and radiocarbon dating, Yiftah’el is slightly younger than Ahihud, nevertheless, it is remarkable that main taxa recovered from the pits in Yiftah’el are also the most abundant legumes found in Ahihud. The economic value of faba bean and lentils in the region is further supported by the preponderance of these two species in the archaeobotanical record of the PPNB sites of Nahal Zippori 3[8] and Nesher Ramla quarry [58].

In addition to the study of the archaeological contexts, the analysis of the archaeobotanical assemblage provides additional information that helps to determine the origin of the seeds and the way in which they entered into the archaeological deposit. Among the aspects to consider, the more relevant ones are *i)* the composition of the archaeobotanical assemblage, *ii)* the state of preservation of the remains.

The first remarkable aspect was that weeds that are common in legumes fields, such as *Phalaris* spp., *Polygonus aviculare*, *Ridolfia segetum*, *Sinapis arvensis*, *Lactuca seriola*, *Avena* spp., *Cuscuta campestris*, *Orobanche crenata* and *Anagallis arvensis* [59, 60] are absent from the archaeobotanical deposit. While we cannot exclude that the small seeds of *Orobanche crenata*, might have escaped the mesh used during sieving, weeds that are larger than the millimeter-sized mesh must have been intentionally removed before being brought to the site.

The archaeobotanical assemblage from L 450 can be considered large and homogenous, since it is made by thousand seeds of a single species. Overall, the state of preservation of the stock is remarkably good, with ~50% of the faba bean showing an intact radicle. This fragile tissue is known to break during the charring, when the seed coat detaches from the cotyledons, and the fact that it is still in place proves that faba beans did not move much after being exposed to fire. Noticeably, none of the seeds shows any sign of germination or parasitic attacks, as we would have expected if spoiled material had been thrown into the pit.

The presence of a single species of edible legume, in a remarkable state of preservation, suggests that stock inside L 450 was meant to be stored in the pit and there it went burnt. Since the seeds are not spoiled, we can exclude that fire was used to clean the pit and we can only speculate about the origin of the fire.

Unlike L 450, the assemblage found in L 398 includes more than one species of legume, which might led one to think that legumes had slipped inside the pit accidentally and that the structure had been used as refuse pit. However, had this been the case, peas or vetchlings on the floors or elsewhere in the site should have been recovered, while they are unique to L 398 [61]. The good state of preservation of the seeds, most of which still present an intact radicle, is another indication that the vegetal material did not roll inside the pit, but rather was burnt *in situ*.

The large quantity of domesticated legumes such as faba bean and, most likely, lentil indicate that these two species were meant for consumption. Furthermore, the lack of spoiled material or segetal plants in the pits is a proof that the stocks of legumes were intentionally stored in the pits. While the economic value of faba bean and lentil is clear, the role of pea and the vetchilings cannot be easily assessed based on the sole context of finding. Insights into the use of such species might come from ethnographic studies and agronomic considerations.

### Crop farming, fodder and storage systems

Besides *Pisum*, whose value as food is indisputable, other legumes such as *Vicia ervilia* and *V*. *narbonensis* are potentially edible (i.e. might have been consumed both by humans and animals). While *Vicia ervilia* and *V*. *narbonensis* are mildly toxic and mostly used as fodder by modern farmers, they are nevertheless consumed by both modern and ancient humans, once their toxicity is eliminated with boiling [62, 63]. Another category recovered at the site is that of the potential fodder (i.e. *Lathyrus hierosolymitanus*, *L*. *inconspicuus*) [64–68]. The presence of fodder species in the site would be justified by the early attempts to domesticate animals developed in the region since the EPPNB [69, 70].

Base on the plant height and seed size [71] we can differentiated between the short-growing species, which reach only 10–50 cm at maturity (i.e. *Lathyrus hierosolymitanus*, *L*. *inconspicuus*, *Lens* sp. *V*. *ervilia*, *V*. *narbonensis*) and the tall-growing species, which are above 50 cm of height at maturity (i.e. *Pisum* sp. and *Vicia faba* L.) (**Table 4**). According to the size of the seeds, we can also differentiate between the small-seed species, which have seeds of diameter or average length below 4 mm (i.e *Lathyrus hierosolymitanus*, *L*. *inconspicuous*, *Pisum* sp. and *Lens* sp.) and medium to large-seed species which are, on average, longer than 4 mm (i.e *Vicia faba* L., *V*. *ervilia*, *V*. *narbonensis*) [71].

**Table 4:** The species found in Ahihud divided according to the type, the plant height and the seed maximum length.

Specie	Type	Plant Height (cm)		Seed Lenght (mm)	
		<50	>50	<4	>4
*Vicia faba*	Actual crop		X		X
*Lens* sp.		x		X	
*Pisum* sp.	Potential edible legumes		X	X	
*Vicia ervilia*		x			X
*Vicia narbonensis*		x			X
*Lathyrus hierosolymitanus*	Potential fodder	x		X	
*Lathyrus inconspicuous*		x		X	

The largest diversity was found in the storage pit L 398, where all sizes of seeds and all categories of legumes.

The presence of three crops (i.e. pea, lentil and faba bean) might suggest that the legumes were farmed together in a regime of mixed cultivation, where each crop was farmed in the same plot. A similar practice has been recorded by ethnographical studies in the northern highlands of Ethiopia in the early 90’s, where pea and faba were grown together to make up for the under-performance of some varieties in certain environmental conditions [72, 73]. Since we do not have conclusive evidence that *Pisum* had been domesticated by the PPNB, it is equally possible that the plant had infested faba bean and lentil fields and was brought to the site as forage.

The presence of short-growing plants, such as *Lathyrus hierosolymitanus*, *L*. *inconspicuus*, *Lens* sp. *V*. *ervilia*, *V*. *narbonensis*, in the assemblage of L 398 suggest that the legumes were harvested close to the ground, which would have allowed the collection of the foliage and the plant stem along with that of the pods. Harvesting legumes by uprooting the plants was popular among the traditional famers of Ethiopia [74], where the legumes were harvested when in a semi-green state to reduce seed loss from pod shatter; the weeds were uprooted as a separate harvest for fodder. The yield was left for one-two days to dry and, after threshing and winnowing, the crop fraction and the fodder were brought home where seeds would be further cleaned by hand before being stored. Storage facilities included clay bins and bamboo baskets sealed with dung, while small quantities were kept in skin bags [74].

The presence of fodder and potentially edible plants in the assemblage of L 398 could be interpreted in one of three different ways. Either the vetchlings and the peas were weeds that infested the crop fields, and they were unintentionally collected during the harvesting, or they were purposely grown together with faba beans and lentils. A third possibility could be that the vetchlings and peas were leftovers of previous harvested and got mixed once the crops were stored in L 398.

The shallow shape of the storage pit L 398 also suggests that some aboveground ephemeral structure, similar to those used by the Ethiopian farmers, was present to accommodate the legumes.

Unlike L 398, L 450, contained almost exclusively the tall-growing plant, medium-large faba bean. The absence of potential fodder and the low frequency of short-growing and small-seed plants in L 450 can be due to three different reasons: *i*) the yield came from a field where the fodder were removed before harvesting; *ii*) the harvesting targeted the pods, or the upper part of the plant, so that short-growing plants would remain on the ground; *iii*) the seeds were sifted before storage [75, 76].

The differences between L 450 and 398, which are based on the diversity of legumes stored and the ‘architecture’ of the storage facility, might reflect two different methods of storing. A thick-walled deep pit containing exclusively crops, such as L 450, could have been more suitable for long-term storing and serve as a communal storeroom for the inhabitants of the site.

A shallow pit, such as L 398, may have been more suitable per short-term storage and serve better for domestic purposes.

The discovery of a large and varied assemblage of legumes in Ahihud offers an unprecedented opportunity to investigate an alternative system of farming based on pulses rather than cereals. Among the sites that present relatively high concentration of legumes, such as Mureybet [77], Dja’de [17], Ghoga Golan [78], Tell Qarassa [18] and M’lefaat [54], only M’lefaat shows percentage of legumes that exceed the 50%, whereas in Ahihud, legumes make more than 90% of the total archaeobotanical assemblage. The only other sites that show a pattern similar to Ahihud are Yiftha’el [56], Nahal Zippori 3 [8] and Nesher Ramla quarry [58], which are few kilometer away from each other and represent different stages of a similar culture.

These sites are close to the area where the wild progenitor of faba bean is known to have existed [7] and are also located within the area of natural distribution of wild lentil and pea [79]. One possibility is that the exploitation of the legumes, that is attested in the area since the Palaeolithic [53], has evolved into cultivation and, finally, into domestication by the time the first villagers settled the Lower Galilee.

### Legumes in the southern Levant: The archaeobotanical findings

Legumes in archaeological sites of the southern Levant have been found from the Middle Palaeolithic (Mousterian) with the discoveries of *Lathyrus inconspicuus*, *L*. *hirosolymitanus*, *Lens* sp., *Vicia narbonensis*, *V*. *ervilia* in Kebara Cave [53] to the present. The earliest remains of *Pisum* (*sativum* subsp. *humile*) are from the ~23.000 yr old site of Ohalo II on the southwestern shore of the Sea of Galilee [80, 81], and later on from the Natufian layers of Hayonim cave [82]. *Lens* sp. is the most common legume in prehistoric times, both in the dry and Mediterranean areas, and has been recovered from several Pre-Pottery Neolithic, Neolithic and Chalcolithic sites in the southern Levant (for details see **Table 5**). While lentil and pea are among the first to appear, being exploited by hunter-gatherers and early dwellers, the faba bean (*Vicia faba* L.) starts to appear in the archaeological record during later periods. The earliest remains of faba date to the Early Natufian and was fond in el-Wad [7]. Later attestation include Iraq ed-Dubb in the Jordan valley [12], Ahihud [3 and this study], Horvat Galili in the Upper Galilee [9], Yiftah’el [4] and Nahal Zippori 3 in the Lower Galilee [3; 8] and in Ain Ghazal in north-western Jordan [83; 13]. Other than the PPNB sites in the Lower Galilee, where large amounts of faba bean were found [3], the faba remains are scarce and there are no data that support the farming or storing of this specific legume. Additional findings of faba bean in prehistoric sites are limited to a few remains in the Pottery Neolithic layers of Nahal Zehora in the Menashe Hills [10], and Sha’ar hagolan in the Jordan valley [11]. The faba bean is regularly identified from the Bronze Age onward, with remains being found in several archaeological sites of the period such as Megiddo [84], Numarya [85], Tell Abu-al Kharaz [86], Tell Ifshar [87], Giv’at Sharet and Tell Miqne [88], Tell Nimrin [89], Timna-Tell Batash [90], Tel Aphek [91] and Pella [92] (**Tables 6** and **7**).

**Table 5:** Summary of the identified legumes found in prehistoric sites of the southern Levant.

	Site	Legume specie							Reference
		*Lens* ssp. (*culinaris/* *orientalis*)	Pisum ssp. (*humile/* *eleatius*)	*Lathurys hierosolymitanus*	*Lathyrus incospicuus*	*Vicia ervilia*	*Vicia faba* sp.	*Vicia narbonensis*	
	**Paleolithic/Epi-palaeolithic/Natufian**								
**31**	Kebara	**x**		**x**	**x**	**x**		**x**	[53]
**39**	Ohalo II	**x**	**x**						[80, 81]
**21**	el-Wad	**x**					**x**		[7]
**23**	Hayonim Cave		**x**						[82]
	**Pre-pottery Neolithic**								
**19**	el-Hemmeh	**x**							[93]
**27**	Iraq ed-Dubb	**x**					**x**		[12]
**63**	Zahrat adh-Dhra'2	**x**							[94]
**26**	Horvat Galili						**x**		[9]
**36**	Nesher Ramla quarry	**x**		**x**			**x**		[58]
**5**	Ahihud	**x**	**x**	**x**	**x**	**x**	**x**	**x**	This study[3]
**62**	Yiftah'el	**x**					**x**		[4]
**35**	Nahal Zippori 3	**x**					**x**		[3; 8]
**29**	el-Jilat 7	**x**				**x**			[12]
**12**	Beidha	**x**				**x**			[12, 95]
**6**	Ain Ghazal	**x**	**x**			**x**	**x**		[13, 83]
**11**	Basta	**x**	**x**			**x**			[96]
**9**	Atlit-Yam	**x**							[97]
**61**	Wadi Fidan C	**x**							[12]
	**Neolithic/Chalcolithic**								
**34**	Nahal Zehora	**x**					**x**		[10]
**42**	Sha'ar hagolan	**x**				**x**	**x**		[11]
**3**	Abu Thawwab	**x**	**x**						[98]
**30**	Jilat 13	**x**							[12]
**46**	Sites in Golan	**x**	**x**						[99]
**44**	Shiqmim I	**x**							[100]
**53**	Tell Abu Matar	**x**							[101]
**2**	Abu Hamid	**x**	**x**						[102]
**39**	Pella	**x**	**x**						[92]
**28**	Jawa	**x**	**x**			**x**			[103]

On the left, the number given to each site in Fig 2.On the right, the reference number is reported in brackets

**Table 6:** Summary of the identified legumes found in protohistoric sites of the southern Levant.

	Site	Legume specie							Reference
		*Lens* ssp. (*culinaris/* *orientalis*)	Pisum ssp. (*humile/* *eleatius*)	*Lathurys hierosolymitanus*	*Lathyrus incospicuus*	*Vicia ervilia*	*Vicia faba* sp.	*Vicia narbonensis*	
	**Bronze Age**								
**10**	Bâb edh-Dhrâ	**x**	**x**						[104]
**32**	Megiddo	**x**					**x**		[84]
**38**	Numayra (Ras an)	**x**							[105]
**55**	Tell el-Hayyat		**x**						[106]
**24**	Hirbet ez-Zeraqon	**x**				**x**			[107]
**37**	Numayra	**x**	**x**			**x**	**x**		[104]
**13**	Beit She'an	**x**				**x**			[108]
**52**	Tell Abu al-Kharaz	**x**				**x**	**x**		[86]
**58**	Tell Ifshar	**x**				**x**	**x**		[87]
**43**	Shiloh	**x**				**x**			[109]
**22**	Giv'at Sharet						**x**	**X**	[88]
**59**	Tell Nimrin	**x**	**x**			**x**	**x**		[89]
**50**	Tel Miqne						**x**		[88]
**60**	Timnah-Tell Batash III	**x**				**x**	**x**		[90]
**47**	Tel Aphek	**x**				**x**	**x**		[91]
**17**	Deir 'Alla		**x**			**x**			[110]
**40**	Pella	**x**				**x**	**x**		[92]

On the left, the number given to each site in Fig 2.On the right, the reference number is reported in brackets

**Table 7:** Summary of the identified legumes found in protohistoric and historic sites of the southern Levant.

	Site	Legume specie							Reference
		*Lens* ssp. (*culinaris/* *orientalis*)	Pisum ssp. (*humile/* *eleatius*)	*Lathurys hierosolymitanus*	*Lathyrus incospicuus*	*Vicia ervilia*	*Vicia faba* sp.	*Vicia narbonensis*	
	**Iron Age**								
**17**	Deir 'Alla	**x**	**x**			**x**			[110, 111]
**32**	Megiddo	**x**				**x**	**x**		[84]
**49**	Tel Keisan					**x**			[112]
**47**	Tel Aphek	**x**	**x**	**x**		**x**	**x**		[91]
**4**	Afula					**x**			[113]
**48**	Tel Hadar					**x**			[114]
**43**	Shiloh	**x**				**x**	**x**		[109]
**51**	Tel Qasile	**x**					**x**		[115]
**40**	Pella	**x**	**x**						[92]
**57**	Tell Hesban	**x**							[116]
**25**	Horbat Rosh Zayit						**x**	**x**	[117]
**56**	Tell el-Husn	**x**				**x**			[118]
**13**	Beit She'an	**x**							[91]
**59**	Tell Nimrin	**x**	**x**			**x**	**x**		[89]
**33**	Motza	**x**							[88]
**8**	Ashkelon	**x**				**x**			[119, 120]
**18**	Dhiban					**x**			[121]
	**Historical periods**	** **	** **	** **	** **	** **	** **	** **	** **
**40**	Pella	**x**	**x**				**x**		[92]
**57**	Tell Hesban	**x**	**x**			**x**	**x**		[116]
**41**	Petra	**x**	**x**			**x**			[122,123]
**20**	el-Lejjun	**x**	**x**						[124]
**15**	Cave of the Spears	**x**	**x**			**x**		**x**	[125]
**1**	Abi'or Cave						**x**		[126]
**14**	Bir Madhkur	**x**				**x**			[127]
**45**	Shivta		**x**						[128]
**7**	Araq el Emir		**x**						[129]
**16**	Cesarea	**x**	**x**			**x**	**x**		[130,131]

On the left, the number given to each site in Fig 2.On the right, the reference number is reported in brackets

## Conclusion

Our findings represent the largest and earliest evidence of intensive farming of legumes in the southern Levant, and show the importance of *Vicia faba* L. as a crop already in the Early Pre-Pottery Neolithic B. The radiocarbon dating of twelve legumes dates the beginning of the process already in11^th^ millennium BP, almost three hundred years earlier than previously thought. The findings provide important evidence for an established agricultural system, based on farming legumes, which developed in Lower Galilee in the Early Pre-Pottery Neolithic B that continued to flourish, at least, until the Middle Pre-Pottery Neolithic B (see Yiftah’el and Nahal Zippori 3).

The discovery of two storage facilities, one exclusively filled with faba beans, and the other with several type of legumes, offers new insights on the agricultural and/or storing practices associated to early farming of founder crops, potentially edible legumes. It is possible the two storage pits were meant for different uses, with faba beans being stocked separately from the other legumes, in the deeper silo, to ensure a supply of the crop over a longer period. A shallow pit, such as L 398, may have been more suitable per short-term storage and serve for domestic purposes.

The results presented in this paper provide new and original insights in the early farming of crop and fodder legumes in Western Asia. Further discoveries might help to improve our understanding of the process of domestication and the role that storage played in the selection of domestication-traits in legumes.
